# Comparative Evaluation of Colistin-Susceptibility Testing in Carbapenem-Resistant Klebsiella pneumoniae Using VITEK, Colistin Broth Disc Elution, and Colistin Broth Microdilution

**DOI:** 10.7759/cureus.65796

**Published:** 2024-07-30

**Authors:** Tessa Antony, Yugadharshini Senthilnathan, Rukmani Madhavakumar, Premika Amudhan, Shruthi Venkataraman, Sanjana Rally, Ravi S Pitani, Ilakkiya Arumugam

**Affiliations:** 1 Microbiology, Sri Ramachandra Institute of Higher Education and Research, Chennai, IND; 2 Community Medicine, Sri Ramachandra Institute of Higher Education and Research, Chennai, IND

**Keywords:** colistin broth disk elution, broth microdilution, colistin resistance, carbapenem resistance, klebsiella pneumoniae (kp)

## Abstract

Purpose: The study aimed to compare the results of colistin-susceptibility testing performed using the automated VITEK system, colistin broth microdilution (BMD), and colistin broth disk elution (CBDE) methods.

Materials and methods: This exploratory study was conducted in a tertiary care center in South India. Carbapenem-resistant *Klebsiella pneumoniae* (n = 49) isolates collected from a clinical microbiology laboratory over six months (March-September 2023) were used for the study.

Results: Among the 49 carbapenem-resistant *Klebsiella pneumoniae* isolates, 42 were found to be susceptible to carbapenem by all three methods. Seven isolates were found to be resistant to colistin using BMD and CBDE methods. Two isolates were incorrectly detected as colistin-susceptible, and one isolate was wrongly categorized as colistin-resistant using the automated VITEK system.

Conclusion: CBDE is a reliable and cost-effective method that can be adopted in the routine microbiology laboratory for colistin-susceptibility testing, as it does not require any specialized equipment or techniques and is 100% consistent with the gold standard BMD method. Although the automated VITEK system is used in most routine microbiological laboratories for antibiotic-susceptibility testing, it cannot be reliably used for colistin-susceptibility testing due to its high error rates (very major error rate of 28.5%; major error rate of 2.4%).

## Introduction

*Klebsiella pneumoniae* is a Gram-negative bacillus belonging to the family Enterobacteriaceae that has the ability to develop resistance to multiple drugs. It is one of the most important pathogens and is associated with both community-acquired and hospital-acquired infections. It is also included in the list of ESKAPE (*Enterococcus faecium, Staphylococcus aureus, Klebsiella pneumoniae, Acinetobacter baumannii, Pseudomonas aeruginosa, Enterobacter spp.*) pathogens that represents a global threat to human health [1]. There has been a surge in reports of infections because of carbapenem-resistant, Gram-negative bacilli in recent years. Dealing with these strains is challenging because of their multidrug-resistant (MDR) nature and their potential to cause nosocomial outbreaks. Resistance to carbapenem is mediated by the presence of carbapenemases, efflux pumps, or decreased porin permeability. Detection of carbapenem resistance can be accomplished by phenotypic methods such as disk diffusion, broth microdilution (BMD), epsilometer test (E test), automated susceptibility testing methods, Carba NP test, matrix-assisted laser desorption/ionization-time of flight (MALDI-TOF), and also by genotypic methods. Antibiotics that can be used to treat these organisms include cefiderocol, polymyxins, aminoglycosides, tigecycline, fluoroquinolones, trimethoprim-sulfamethoxazole, and beta-lactam/beta-lactamase inhibitors such as ceftazidime-avibactam, imipenem-cilastatin-relebactam, or meropenem-vaborbactam [2].

Even though polymyxins were introduced into clinical practice in the 1950s, they were not preferred for use in patients because of the neurotoxic and nephrotoxic side effects and the availability of safer alternatives [3]. Because organisms developed resistance to these safer alternatives, polymyxins are more frequently employed now as last-resort therapy for a variety of multidrug-resistant microorganisms. These antibiotics have potent bactericidal action against a variety of Gram-negative microbes. They have significant effects against most members of the Enterobacteriaceae family, which includes *Escherichia coli*, *Klebsiella *spp., *Enterobacter *spp., *Citrobacter *spp., and some of the common nonfermentive Gram-negative bacilli such as *Pseudomonas aeruginosa*, *Acinetobacter baumannii*, and *Stenotrophomonas maltophilia*. During the past few decades, the emergence of polymyxin-resistant isolates has been increasing worldwide because of their widespread use both clinically and nonclinically, such as in veterinary growth promotion and the use of nonstandardized methods of in vitro colistin-susceptibility testing [4,5].

Colistin (polymyxin E) is a large polycationic peptide that attaches to the negatively charged cell of Gram-negative bacteria and disrupts its outer membrane, resulting in cell death. Colistin resistance can be intrinsic or acquired. Intrinsic resistance to this antibiotic is seen in some Gram-negative bacilli such as *Serratia* spp., *Proteus *spp., and *Burkholderia *spp.

Resistance to colistin can be mediated by alteration of the lipopolysaccharide (LPS) of the bacterial outer membrane, leading to a reduction in net negative charge, overexpression of efflux-pump systems, overproduction of capsular polysaccharide, and rarely by the production of enzyme colistinase [6]. The genetic basis of resistance can be chromosomal or mediated by plasmids. Modification of the LPS commonly occurs by the addition of phosphoethanolamine, which results in a reduction of the net negative charge and prevents the binding of the cationic antibiotic colistin. The addition of phosphoethanolamine is done by a phosphoethanolamine transferase enzyme encoded by the pmr or mcr gene [7]. Resistance can occur because of point mutation and/or genetic disruption in two-component regulatory systems, pmrAB and phoPQ, that control the expression of genes encoding the transferase enzyme and inactivation of mgrB, a regulatory transmembrane protein that controls the kinase activity of phoQ in phoPQ [8]. Being plasmid-mediated, the mobile colistin resistance gene (mcr-1) and its variants (mcr-2 to mcr-8) can readily disseminate among clinical pathogens [9].

The clinical challenges of colistin use include issues related to their drug toxicity, limited pharmacokinetic-pharmacodynamic data, lack of clinical outcomes, and lack of adherence to standard guidelines for antimicrobial susceptibility testing (AST) of the drug. According to the Clinical and Laboratory Standards Institute (CLSI) and European Committee on Antimicrobial Susceptibility Testing (EUCAST) guidelines, BMD without surfactant is the reference method for testing colistin. Although BMD is an accurate method for colistin minimum inhibitory concentration (MIC) determination, it is a difficult test to perform in routine clinical microbiology laboratories. Neither CLSI nor EUCAST recommends utilizing disk and gradient diffusion methods for testing colistin because of unacceptably high error rates.

Automated methods for determining the MIC of colistin are carried out in clinical microbiology laboratories because they are less cumbersome than the conventional BMD. VITEK 2 (bioMérieux, Lyon, France), MicroScan Walkaway (Dade-Behring MicroScan, Deerfield, Illinois), and Phoenix system (BD Diagnostic Systems, Franklin Lakes, New Jersey) are automated platforms that have been widely used across the world. These instruments use optical systems for determining bacterial growth and antimicrobial susceptibility.

CLSI advocated two MIC-based methods in 2020 for testing colistin susceptibility in Enterobacterales and *P. aeruginosa*. Both methods, colistin broth disk elution (CBDE) and the colistin agar test can easily be performed in a normal microbiology laboratory. In this study, we compared the colistin-susceptibility test results of automated VITEK 2 and CBDE to the colistin BMD among carbapenem-resistant *Klebsiella pneumoniae* isolates. Because colistin resistance was found to be stronger among the clinical *Klebsiella pneumoniae* isolates, we selected this species for the study.

## Materials and methods

This exploratory study was conducted in a tertiary care teaching hospital in South India after obtaining approval from the Institutional Ethics Committee at Sri Ramachandra Institute of Higher Education and Research (IEC-NI/22/DEC/85/129 dated February 21, 2023). The study included 49 nonrepetitive, carbapenem-resistant *Klebsiella pneumoniae* (CRKP) isolates from various samples (pus, urine, respiratory, and blood samples) collected in the microbiology laboratory.

We performed aerobic bacterial cultures from all the clinical samples and subjected the isolated bacterial colonies to Gram stain and matrix-assisted laser desorption ionization-time-of-flight mass spectrometry (MALDI-TOF MS) by VITEK MS (bioMérieux, France) for species-level identification. We performed AST for all isolates using the VITEK 2 Gram-negative bacilli N405 panel according to the manufacturer’s instructions. Carbapenem resistance in *Klebsiella pneumoniae* isolates was detected by MIC using CLSI guidelines. If the isolate was found to be resistant to any of the carbapenems (ertapenem MIC ≥ 2 μg/mL, imipenem MIC ≥ 4 μg/mL, meropenem MIC ≥ 4 μg/mL), it was assumed to be carbapenem-resistant.

All 49 CRKP isolates were subjected to colistin susceptibility testing by BMD, CBDE, and automated VITEK 2 AST using the Gram-negative bacilli N405 panel.

Broth microdilution

BMD was performed using untreated, 96-well polystyrene plates in which colistin concentrations ranged from 0.25 to 16 μg/mL. We used the standard operating protocol issued by the National Programme on Antimicrobial Resistance Containment, National Centre for Disease Control, India, February 2021 [10]. Cation and pH-adjusted Mueller-Hinton broth (MHB) (HiMedia, Mumbai, India) was used for performing BMD. Bacterial suspensions were then inoculated into each well to achieve a final concentration of 10⁵ colony forming units (CFU)/mL. To monitor the healthiness of isolates and media sterility, a drug-free column and an uninoculated column with media were included in each assay.

Preparation of Colistin Primary Stock Solution Formulation

The reference standard powder formulation used was colistin sulfate (30,000 units/mg or 30 units/μg) as per CLSI standards. The potency of the colistin powder available in colistin sulfate salt mentioned in the certificate of analysis (CoA) was 19,000 units/mg (Sisco Research Laboratory, Mumbai, India). To derive the potency of the colistin powder in reference to the pure agent in μg/mg, we divided the given potency in units/mg (as given in the CoA of the colistin powder being used) by the reference potency of 30 units/μg and got a value of 633 μg/mg. We prepared a stock solution of 1000 μg/mL (1 mg/mL) by adding 10 mg of the colistin sulfate powder with a potency of 633 μg/mg to 6.63 mL of autoclaved distilled water (6330 μg/6.63 mL).

The primary stock solutions were dispensed in volumes of 200 μL in sterile 2 mL cryovials/cryotubes and stored at −80 °C. From the primary stock solution, the working stock solution was prepared, which had four times the final drug concentration. We prepared a working stock solution of 64 μg/mL by adding 64 μL from the primary stock solution to 936 μL of autoclaved MHB medium in another microcentrifuge tube (MCT). We made serial dilutions from the working stock solution by adding 500 μL from the 64 μg/mL to 500 μL of MHB medium in an MCT. Further, we made twofold serial dilutions (in seven MCTs containing 500 μL MHB) as shown in Table 1 to achieve final drug concentrations of 16 μg/mL, 8 μg/mL, 4 μg/mL, 2 μg/mL, and so on.

**Table 1:** Preparation of specific colistin concentrations in microtiter wells. MCT: microcentrifuge tube, MHB: Meuller-Hinton broth.

	1	2	3	4	5	6	7	8
MCT	936 μL MHB + 64 μL primary stock solution	500 μL MHB +500 μL MHB with 64 μg/mL colistin from MCT-1	500 μL MHB +500 μL MHB with 32 μg/mL colistin from MCT-2	500 μL MHB +500 μL MHB with 16 μg/mL colistin from MCT-3	500 μL MHB +500 μL MHB with 8 μg/mL colistin from MCT-4	500 μL MHB +500 μL MHB with 4 μg/mL colistin from MCT-5	500 μL MHB +500 μL MHB with 2 μg/mL colistin from MCT-6	500 μL MHB +500 μL MHB with 1μg/mL colistin from MCT-7
Colistin concentration (μg/mL) in MCT	64	32	16	8	4	2	1	0.5
Final colistin concentration (μg/mL) in microtiter wells	16	8	4	2	1	0.5	0.25	0.125

Preparation of 96-Well Microtiter Plate for Colistin BMD

The total volume of each well was 100 μL. We added 50 μL of MHB in Wells 1 to 8, 75 μL of MHB to Well 11 as growth control, and 100 μL of MHB in Well 12 for sterility control. We added 25 μL of the different concentrations of a working stock solution of antibiotics from the first to the eighth wells.

Inoculum Preparation

We made saline suspensions in tubes from three to five well-isolated colonies of the same morphological type from an 18-24-hour culture plate. The turbidity was adjusted to 0.5 McFarland turbidity standard (approximately 1.5 x 10⁸ colony forming units (CFU)/mL) using a densitometer. We added 10 μL of this solution to 740 μL of autoclaved MHB medium to further dilute the suspension 1:75 times.

To each of the wells in Columns 1-8, 11, and 12, which already contained 75 μL (50 μL MHB + 25 μL antibiotic), we added 25 μL from this diluted suspension to yield a bacterial concentration of approximately 5 x 10⁴ CFU/well. Within 15 minutes of adding the inoculum, the microtiter plates were incubated at 35°C ± 2°C for 18-20 hours in an ambient air incubator. To prevent drying, each microtiter tray was sealed with a plastic cover before incubating. For quality control (QC), the American-type culture collection (ATCC) strains ATCC 25922 *Escherichia coli *and ATCC 27853 *Pseudomonas aeruginosa *were also inoculated with the test isolates.

**Figure 1:**
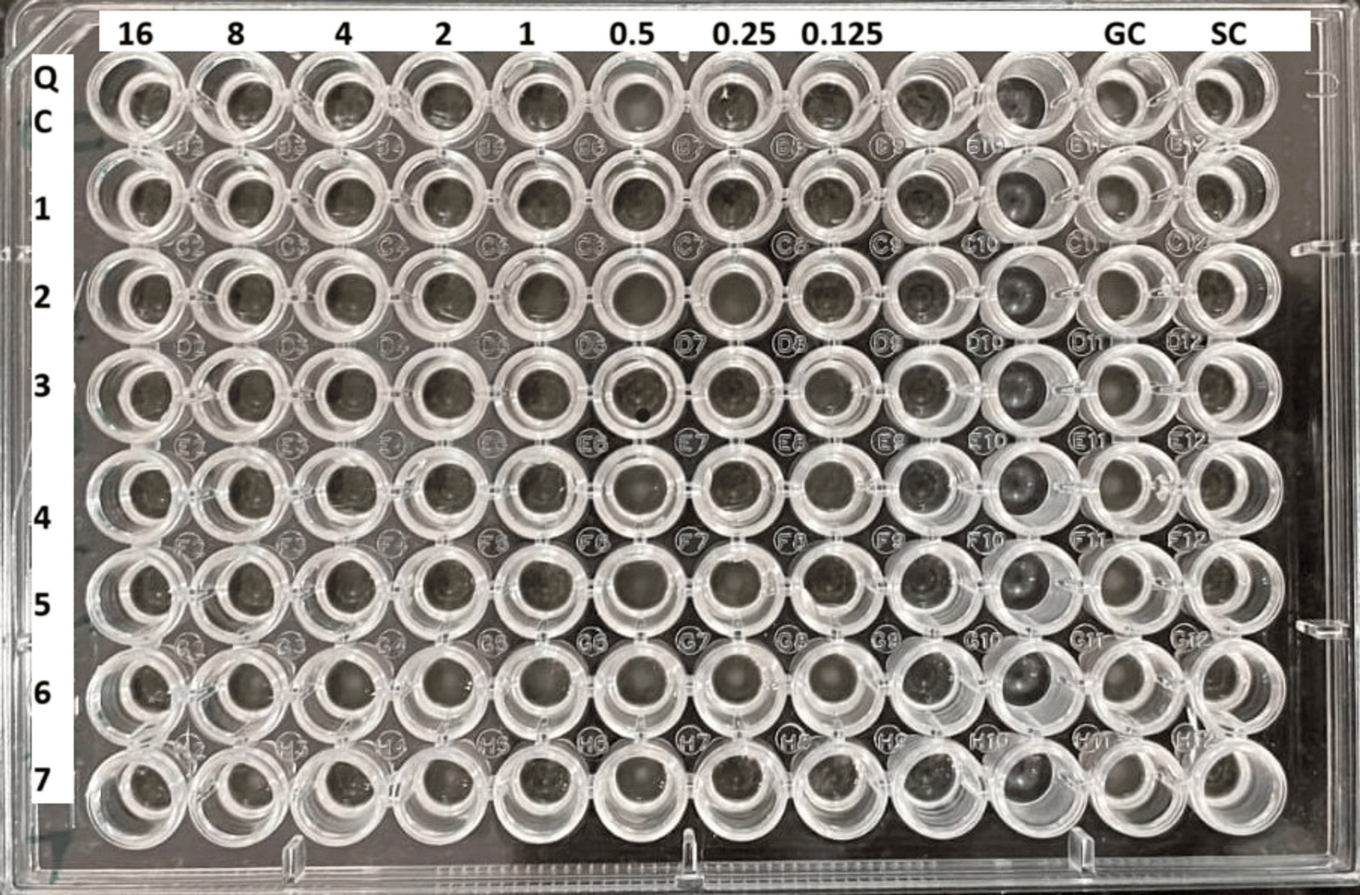
Colistin broth microdilution. Quality control strain: ATCC *Escherichia coli *29522 minimum inhibitory concentration (MIC) 1 μg/mL, carbapenem-resistant *Klebsiella pneumoniae *(CRKP): 1, CRKP1; 2, CRKP 2; 3, CRKP 3 (MIC ≤ 0.125 μg/mL); 4, CRKP 4; 5, CRKP 5 (MIC 1 μg/mL); 6, CRKP 6; 7, CRKP 7 (MIC 16 μg/mL). GC: growth control, SC: sterility control.

We next determined the broth microdilution end points. The lowest concentration of colistin that completely inhibited the growth of the organism in the microdilution wells, as detected by the unaided eye, was taken as the MIC of colistin. The test is valid if acceptable growth (definite turbidity or button) has occurred in the growth-control well and no turbidity has occurred in the sterility-control well. MIC results for the quality control strains were found to be within the acceptable limits (0.5-2 μg/mL for ATCC *Escherichia coli* 25922 and 0.5-4 μg/mL for ATCC *Pseudomonas aeruginosa* 27853) before interpreting the test results of the isolates (Figure 1).

Colistin broth disk elution (CBDE)

The CBDE employs commercially available colistin disks of 10 μg potency. Cation-adjusted MHB (10 mL) was added to four borosilicate tubes that were labeled as 1, 2, and 4 μg/mL and control. To the first three tubes, 10 μg colistin sulfate disks (HiMedia, India) were added using aseptic techniques to obtain final concentrations of 1 μg/mL (one disk in 10 mL), 2 μg/mL (two disks in 10 mL), and 4 μg/mL (four disks in 10 mL). No disks were added to the control tube (0 μg/mL; 0 disks in 10 mL).

**Figure 2:**
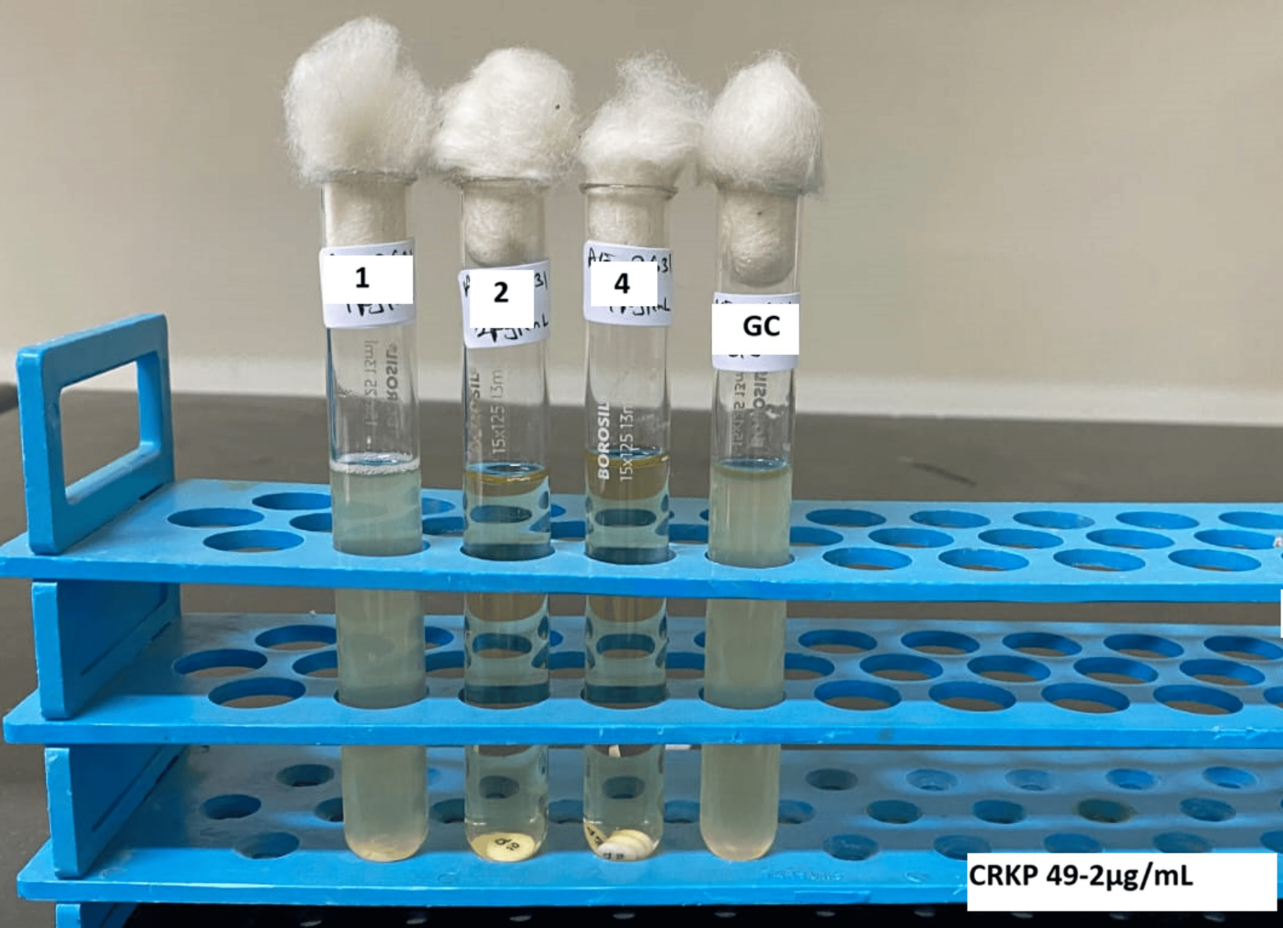
Colistin broth disk elution test showing minimum inhibitory concentration of 2 μg/mL for test isolate. CRKP: carbapenem-resistant *Klebsiella pneumoniae.*

After allowing colistin to elute from the antibiotic disks (> 30 minutes and < 60 minutes) at room temperature, the tubes with the antibiotic disks were gently vortexed. We prepared standardized inoculum (50 μL of 0.5 McFarland standard) for each test isolate by picking three to five colonies from fresh (18-24 hours) nonselective agar plates and adding them to all four tubes to attain a final inoculum concentration of approximately 7.5 x 10⁵ CFU/mL per tube. After vortexing, all the tubes were incubated at 33-35°C in ambient air for 16-20 hours. The presence of noticeable turbidity in the growth control tube was a prerequisite for test validity. MIC was taken as the lowest concentration that completely inhibited the growth of the test isolate. MIC of the QC strain *Pseudomonas aeruginosa *ATCC 27853 was within acceptable limits (≤1-4 μg/mL) before the results of the test isolates were interpreted (Figure 2). In addition to the recommended QC strain, ATCC *Escherichia coli *25922 was also tested by the CBDE method.

Colistin MIC by VITEK 2

Colistin MICs were determined using the commercial VITEK 2 system with the AST N405 panel, according to the manufacturer’s instructions. The categories used for the interpretation of colistin-susceptibility testing according to CLSI and EUCAST guidelines for Enterobacteriaceae are depicted in Table 2 [11]. The EUCAST guidelines have only susceptible (S) and resistant (R) categories, whereas CLSI guidelines have intermediate (I) and resistant (R) categories. In the EUCAST guidelines, the MICs of the drug are put in brackets, which signifies that it should not be used as monotherapy unless used in infections in which high exposure can be achieved at the site of infection [12].

**Table 2:** Colistin minimum inhibitory concentration breakpoints Colistin interpretative breakpoints according to the Clinical Laboratory Standards Institute (CLSI) and the European Committee on Antimicrobial Susceptibility Testing (EUCAST). S: susceptible, I: intermediate, R: resistant.

CLSI (MIC in μg/mL)			EUCAST (MIC in μg/mL)	
S	I	R	S	R
-	≤2	≥4	≤2	>2

Statistical analysis

For comparison among the different methods of testing, the colistin MIC results were truncated to the smallest range of the methods being compared. To assess the performance of MIC testing of colistin using VITEK AST and CBDE with reference to BMD, we evaluated essential agreement (EA) and categorical agreement (CA). We identified categorical disagreements classified as very major errors (VMEs) and major errors (MEs) [9].

## Results

A total of 49 CRKP samples were isolated from urine (n = 30), blood (n = 7), pus (n = 11), and bronchial wash (n = 1) over a period of six months (March-September 2023) in the microbiology laboratory. Resistance to colistin among CRKP isolates was 14.3% (7/49) by the gold standard BMD method from the following samples: urine (4), pus (2), and bronchial wash (1). In vitro resistance of the test isolates to other antibiotics was also found to be very high: ciprofloxacin (100%; 49/49), gentamicin (83.7%, 41/49), amikacin (6.1%, 3/49), tigecycline (6.1%, 3/49), and fosfomycin (51%, 25/49).

EA was defined as the percentage of test results that were within ±1 log2 dilution of reference BMD MIC results. CA was defined as the percentage of test results (susceptible or resistant) that were consistent with reference BMD interpretation categories. If the isolate was resistant in BMD and susceptible in the test method, it was considered a VME (false susceptible result). If the isolate was susceptible in BMD but resistant in the test method, it was considered an ME (false resistant result). VMEs were calculated using the number of isolates resistant in BMD as the denominator. ME rates were calculated using the number of isolates susceptible in BMD as the denominator. For the calculation of ME and VME results of colistin susceptibility testing in the present study, "I" of CLSI was considered equivalent to "S" of the EUCAST guidelines.

CBDE Compared to BMD

EA was 100% (49/49) between the BMD and CBDE methods, and CA was 100% (49/49). VME and ME were 0%, as all the isolates that were resistant in BMD were also found to be resistant in CBDE, and all the isolates that were susceptible in BMD were found to be susceptible in CBDE (Table 3).

**Table 3:** Comparison of minimum inhibitory concentration (MIC) of test isolates by broth microdilution (BMD) and colistin broth disk elution (CBDE) S: susceptible (≤2 µg/mL), R: resistant (>2 µg/mL).

CBDE		Isolates with colistin MIC (μg/mL) by BMD			
		S		R	
Interpretation	MIC in μg/mL	≤1	2	4	> 4
S	≤1	34	1	-	-
	2	5	2	-	-
R	4	-	-	2	1
	> 4	-	-	-	4

VITEK compared to BMD: EA was 93.9% (46/49) and CA 93.9% (46/49) between BMD and VITEK methods (Table 4). VME was 28.5% (2 ("S" by CBDE and "R" by BMD)/7) and ME 2.4 % (1("R" by CBDE and "S" by BMD)/42).

**Table 4:** Comparison of minimum inhibitory concentration (MIC) of test isolates by broth microdilution (BMD) and VITEK 2 S: susceptible (≤2 µg/mL), R: resistant (>2 µg/mL).

VITEK		Isolates with colistin MIC (μg/mL) by BMD			
		S		R	
Interpretation	MIC in μg/mL	≤1	2	4	> 4
S	≤1	38	3	-	1
	2	-	-	-	1
R	4	-	-	1	1
	> 4	1	-	1	2

## Discussion

Colistin is one of the foremost drugs used in the treatment of extensively drug-resistant Gram-negative bacteria. Combination therapy of colistin with other drugs such as carbapenems, sulbactam, tigecycline, or aminoglycosides is preferred to monotherapy. MICs obtained from testing colistin predict MICs to polymyxin B and vice versa because colistin and polymyxin B are considered equivalent agents. Even though accurate reports of global colistin resistance were not found, according to the mobilized colistin resistance gene study (1980-2018), the global prevalence of mcr was found to be 4.7%. The rate of in vitro colistin resistance among *Klebsiella pneumoniae* is documented at 0.2%-0.7% according to the National Antimicrobial Resistance Survey Network India Annual Report 2023 [13]. In the present study, the resistance rate was 14%, which may be because colistin susceptibility testing was performed only in carbapenem-resistant isolates. Sujatha et al. also reported similar colistin resistance rates (11%) among CRKP isolates [14].

The results of the comparison of colistin-susceptibility testing between VITEK and BMD were consistent with the results obtained in the study conducted in Singapore in 2017 by Chew et al., with an EA of 93.4%, a CA of 88.2%, and VME of 36% [15]. The values obtained were slightly different from the study done in 2018 in India by Butta et al., with an EA of 87.3%, a CA of 89.3%, and VME and ME rates of 8% and 2.3%, respectively [16]. Khurana et al. in India (2019) showed a CA of 88% with 10% VMEs and 1% MEs when the results of VITEK 2 were compared with the reference BMD for 910 Gram-negative isolates [17]. Lo-Ten-Foe et al. in the Netherlands showed a high level of agreement of VITEK 2 with BMD; however, VME and ME rates were not stated in the study [18]. Tan and Ng in Singapore showed a VME of 26% when testing Enterobacteriaceae by VITEK 2 compared with the reference colistin agar test [19]. For a new commercial test to be acceptable compared with the reference method, it should have CA and EA of ≥ 90%, VME of <1.5%, and ME of < 3% as per CLS-recommended performance standards [20]. Although according to the current study, EA and CA are >90% and ME < 3%, VITEK results cannot be accepted because VME is 28.5%, which is far above the required standards. The study results have corroborated the findings of earlier studies which state that VITEK may not be a reliable detector of colistin MIC in *Klebsiella pneumoniae*. The high rates of VME may also be due to the lower number of resistant isolates (denominators) by BMD. These results can be further confirmed by conducting a multicentric study with more samples.

CBDE is a CLSI-approved assay for testing colistin susceptibility in Enterobacterales and *Pseudomonas aeruginosa* that can easily be included in routine testing in microbiology laboratories. A two-site evaluation study conducted in Los Angeles and Baltimore in the United States in 2019 by Simner et al. reported CA and EA of 100% with no VME or ME when comparing the results of CBDE to BMD [9]. This was consistent with our study findings. Other studies have reported slightly different findings. The study conducted in India in 2021 by Sujatha et al. showed CA of 98% and VME of 2% [14]. Humphries et al. also reported a similar CA of 98.6% for Enterobacterales with 2.5% VME and 0% ME [21]. In the study by Dalmolin et al. in two research centers in Brazil from 2013 to 2017, CA of 91.18%, EA of 95.9%, ME of 16%, and VME of 4.65% were noted [22].

The findings from this study imply that microbiologists should carefully choose the method available for colistin susceptibility testing. Colistin resistance is prevalent among CRKP isolates, which should make the testing process and infection control practices more stringent. The limitations of the present study include the limited sample size, non-inclusion of *Pseudomonas aeruginosa *and Enterobacterales other than *Klebsiella *spp., and the single-center nature of the study. For routine QC of CBDE, only ATCC *Pseudomonas aeruginosa* 27853 was used because the other recommended strain of *Escherichia coli *BAA-3170 was not available. However, the results of the CBDE and BMD were in 100% agreement with each other. Further molecular testing of the isolate would have helped us understand the mechanisms of colistin resistance. This would aid us in finding the reason for the difference in MIC values obtained in VITEK, which may be able to provide the reason for the incorrect results obtained in VITEK 2 compared to BMD.

## Conclusions

The study was conducted on clinical isolates of *Klebsiella pneumoniae* that were multidrug-resistant and are the usual isolates for which the antibiotic colistin may be prescribed. Although the study was conducted on a limited pool of CRKP isolates from patient samples, CBDE was found to be a reliable method that can be easily performed in the microbiology laboratory. Even though most of the automated microbiology laboratories utilize VITEK 2 for polymyxin or colistin susceptibility testing, the results obtained were found to be unreliable. This warrants a multicentric study with a larger number of clinical isolates from Enterobacterales and *Pseudomonas aeruginosa*, for which CBDE has been approved by CLSI.
